# Periodic fever syndromes: beyond the single gene paradigm

**DOI:** 10.1186/s12969-019-0324-7

**Published:** 2019-05-14

**Authors:** Clara Westwell-Roper, Iwona Niemietz, Lori B. Tucker, Kelly L. Brown

**Affiliations:** 1 Division of Rheumatology, Department of Pediatrics, University of British Columbia, Vancouver, Canada; 2 Department of Microbiology & Immunology, University of British Columbia, Vancouver, Canada; 3 BC Children’s Hospital Research Institute, Vancouver, Canada; 4 BC Children’s Hospital, K4-124 4480 Oak St, Vancouver, BC V6H 3N1 Canada

**Keywords:** Hereditary autoinflammatory diseases, Familial Mediterranean fever, Biological therapy, Interleukin-1, Pyrin, Genetic association studies

## Abstract

Familial Mediterranean fever (FMF) is the most common monogenic autoinflammatory disease in Canada and is characterized by a clinical syndrome of episodic inflammatory symptoms. Traditionally, the disease is defined by autosomal recessive inheritance of *MEFV* gene variants, yet FMF also not uncommonly manifests in individuals with only one identified disease-associated allele. Increasing availability and affordability of gene sequencing has led to the identification of multiple *MEFV* variants; however, they are often of unknown clinical significance. Variants in other genes affecting overlapping or distinct inflammatory signaling pathways – together with gene-environment interactions including epigenetic modulation – likely underlie the significant genetic and phenotypic heterogeneity seen among patients with this disease. We review recent evidence of the expanding spectrum of FMF genotype and phenotype and suggest that current drug funding schemes restricting biologic agents to patients with homozygous mutations have not kept pace with our biological understanding of the disease.

## Background

Many heritable monogenic autoinflammatory diseases present with recurrent fever episodes, often beginning in childhood. These disorders – characterized by clinical and biologic evidence of inflammation in the absence of antigen-specific immunity – include Familial Mediterranean fever (FMF), Tumour necrosis factor receptor-associated periodic syndrome (TRAPS), Cryopyrin-associated periodic fever syndromes, and mevalonate kinase deficiency or hyperimmunoglobulin D syndrome [1]. FMF is the most common monogenic autoinflammatory disease worldwide and in Canada [2], characterized by recurrent, self-limited episodes of fever and inflammation involving serous and synovial membranes. Inherited in an autosomal recessive fashion, its prevalence is highest among populations of Middle Eastern and Mediterranean origin, and is variable in other populations [3]; the exact prevalence of this condition is uncertain. The diagnosis is based on clinical criteria (Table 1) and further supported by genetic testing for disease-associated variants in the *MEFV* gene [4, 5]. *MEFV* encodes pyrin, a cytoskeleton-associated protein that senses perturbations in intracellular homeostasis such as microbial inactivation of Rho GTPases [6]. Its association with apoptosis-associated speck-like protein (ASC) leads to activation of a multiprotein inflammasome complex and downstream production of the potent pro-inflammatory and pyrogenic cytokine interleukin-1β (IL-1β) by neutrophils, monocytes, dendritic cells, and synovial fibroblasts. Recent data suggest a key role for the pro-inflammatory cytokine tumour necrosis factor-α (TNF- α) in modulation of pyrin expression and inflammasome activation [7]. However, pyrin also facilitates autophagic degradation of other inflammasome components, underscoring the complexity of this protein’s function. Although there has been some controversy as to whether disease-associated mutations represent loss of an inhibitor or gain of pro-inflammatory function, data from mutant knock-in and pyrin-deficient mice suggest that at least some mutant alleles are associated with a gain-of-function for pyrin [8] and a reduced inflammasome activation threshold [9].

**Table 1:** Clinical criteria for the diagnosis of FMF

Tel Hashomer clinical criteria (3)	
Diagnostic criteria:	One or more major signs; or Two minor signs; or One minor and five supportive criteria
Major criteria:	≥3 attacks of the same type, with rectal temperature ≥ 38 **°**C, lasting 12–72 h, as well as: Peritonitis Pleuritis or pericarditis Monoarthritis (hip, knee, ankle) Fever alone
Minor criteria:	Incomplete attacks involving 1 or more of abdomen, chest, joint Exertional leg pain Favourable response to colchicine
Supportive criteria:	Family history of FMF Appropriate ethnic origin Age < 20 yr. at disease onset Features of attacks: severe; spontaneous remission; symptom-free intervals; transient inflammatory response with abnormal WBC, ESR, SAA, or fibrinogen; episodic proteinuria/hematuria; unproductive laparotomy; parental consanguinity
Yalcinkaya clinical criteria for children (4)	
Requires ≥3 attacks with ≥2 of the following:	
	Fever with axillary temperature > 38 °C lasting 6–72 h Abdominal pain lasting 6–72 h Chest pain lasting 6–72 h Oligoarthritis lasting 6–72 h Family history of FMF

Recent work based on the Eurofever registry - a large international registry of autoinflammatory diseases - has proposed new provisional clinical classification criteria with high sensitivity and specificity based on a gold standard defined by the presence of two *MEFV* mutations [10]. The criteria include fever episodes lasting less than two days, with accompanying symptoms of chest pain and/or abdominal pain together with Eastern or North Mediterranean ethnicity. Patients should not have aphthous stomatitis, urticarial rash, or enlarged cervical lymph nodes, and episodes may not last more than 6 days [10]. While these criteria remain provisional, other published classification criteria (Table 1) have been developed based on expert opinion and description of clinical manifestations in populations of limited ethnic diversity; the overlap among clinical features has led to low performance when applied to patients with different autoinflammatory diseases [10].

Given that there are overlapping symptoms among FMF and a number of polygenic autoinflammatory diseases – including periodic fever, aphthous stomatitis, pharyngitis and adenitis (PFAPA) syndrome, systemic-onset juvenile idiopathic arthritis (sJIA), and Behçet disease – it is often challenging to make a purely clinical diagnosis of FMF. This is particularly the case in regions such as North America where FMF is rare and may be milder or present atypically [11, 12]. Some patients may also be mistakenly diagnosed with autoinflammatory or autoimmune syndromes that have some overlapping clinical features, including Behcet disease, systemic lupus erythematosus, or rheumatic fever [11].

Moreover, interpretation of genetic testing is challenging in patients with an FMF syndrome or a clear inflammatory phenotype but only one *MEFV* mutation of uncertain significance. Consensus guidelines suggest that while the diagnosis relies on clinical judgment, another periodic fever syndrome (PFS) should be considered in this case [13]. Further studies are needed to validate data from combined molecular and clinical analysis in order to understand the effects of specific genetic variants [10]. As emphasized by a recent systematic review [12], there is wide clinical variability among individuals with an FMF phenotype that is only partially explained by allelic heterogeneity.

The aim of this review is to describe the difficulties faced in clinical settings in making a diagnosis of FMF, or other genetically defined autoinflammatory diseases, in the face of patients with clinical disease and genetic mutations that are defined as ‘uncertain’. The use of patient databases to advance understanding, particularly in genetically mixed populations, and the implications to treatment accessibility when diagnoses are undefined, are discussed.

## Interpreting allelic variants of uncertain significance

Over 60 disease-associated mutations have been identified in *MEFV*, most of which are missense changes and cluster in exons 2 and 10. Common pathogenic mutations include M694 V, M680I, M694I, M694 V, V726A; the online registry INFEVERS provides a comprehensive registry (https://infevers.umai-montpellier.fr/). A subset of these, including E148Q in exon 2 and four mutations in exon 10 (M694 V, M694I, V726A, M680I), account for up to 80% of FMF cases [14]. Whether variants of uncertain significance such as E148Q cause the FMF phenotype remains controversial; because of its high carrier frequency, E148Q is often described as a polymorphism rather than a disease-causing mutation [15]. However, several reports have described patients homozygous for E148Q who have an FMF phenotype and may respond to colchicine [16]. In addition, recent data suggest a spectrum of clinical phenotypes with fewer symptoms, milder disease, and potentially older age at onset [17] in patients with E148Q or V726A variants compared to other mutant alleles [18]. Moreover, individuals who carry one uncertain variant together with a single clearly pathogenic mutation such as M649 V often present with classic FMF [19]. There is currently little available functional data to aid in evaluating the pathogenicity of variants of uncertain significance; indeed, non-confirmatory genetic results may lead to overestimation of the relevance of some of these variants particularly as more are identified through next-generation sequencing. Ongoing observational trials, such as Familial Mediterranean Fever and Related Disorders: Genetics and Disease Characteristics (NCT00001373) and Phenomics in Autoimmune and Inflammatory Diseases (NCT02466217), incorporating functional biological assays to elucidate the functional consequences of genetic variations on the molecular level (i.e. by demonstrating that genetic alterations drive pro-inflammatory signalling), may shed further light on the relationships between gene variants and phenotypes.

## Moving beyond *MEFV* genotype

The presence of two pathogenic *MEFV* mutations *in trans* (i.e., on both chromosomes, as in a homozygote or compound heterozygote) confirms the diagnosis of FMF. However, data from the Eurofever project show that while 55% of patients with a clinical diagnosis of FMF have two *MEFV* mutations, 31% of patients have only one [20]. Indeed, heterozygotes constitute approximately 24–34% of the patient population in most studies, and full sequencing of the entire coding region infrequently reveals additional mutations [21]. The auto-inflammatory disease clinical registry of the BC Children’s Hospital in Vancouver, BC, an initiative modeled on the Eurofever database, currently has 65 enrolled patients with a clinically defined diagnosis of a PFS, of whom 56 have been assessed for *MEFV* genotype by Sanger sequencing across the ten coding exons of the *MEFV* gene. Eight of these patients meet clinical criteria for FMF [22], with ethnicities including West Asian (3 patients), Eastern European/Ashkenazi Jew (2 patients), Irish/Northern European/French, Chinese/Irish/English/Scottish, and Arab. Three have two *MEFV* variants (heterozygotes, with allele variants either in *cis* or *trans*) and five have only one *MEFV* variant (of these, only one is homozygous). *MEFV* variants were also identified in one patient with PFAPA, five patients with unclassified PFS, and one patient with TRAPS (Table 2). These data suggest that our population of patients with an FMF phenotype is genetically diverse, and that – in our Canadian experience – patients with a classic presentation together with typical genetic testing are rare.

**Table 2:** Patients enrolled in the CAN-Fever registry followed over a 6-month period at British Columbia Children’s Hospital with a diagnosis of periodic fever syndrome associated with an *MEFV* mutation

Diagnosis	MEFV allele	Variant
FMF	M694 V	Heterozygous
	K695R	Heterozygous
	P369S/ R408Q^a^ + *TNFRSP1A variant*	Heterozygous
	P369S/ R408Q^a^	Heterozygous
	M694 V	Heterozygous
	K695R	Heterozygous
	M694 V/ R761H	Compound heterozygous
	M694 V + *AP4M1 variant*	Homozygous
Unclassified	P369S^a^/ R408Q^a^	Compound heterozygous
	E148Q^a^/ L110P^a^	Heterozygous
	E148Q^a^	Heterozygous
	E148Q^a^ + *TNFRSP1A variant*	Heterozygous
	E148Q^a^	Heterozygous
PFAPA	V469A^a^	Heterozygous
TRAPS	E148Q^a^	Heterozygous

^a^Variants of unknown significance

The contribution of other genetic or environmental factors is supported by the observation that up to 10–20% of patients with clinical FMF do not have any predictive pathogenic *MEFV* variants [23]. Studies of mono- and dizygotic twins have identified significant effects of both modifying genes and environmental factors on clinical phenotype in siblings with *MEFV* variants [24]. For example, alleles in other genes including MHC class-I polypeptide-related sequence A (*MICA*) and serum amyloid A-1 protein (*SAA1*) are associated with a severe FMF phenotype and increased susceptibility to amyloidosis [25]. Recent reports have described patients who are compound heterozygotes for mutations in other known PFS genes (*CIAS1* and *TNFRSF1A*) [26, 27], suggesting that a spectrum of atypical clinical presentations may be possible with various combinations of allelic variants. In fact, a substantial proportion of patients meeting clinical criteria – up to 40% in a recent study – have none of the 12 most common *MEFV* mutations [28]. This is a far greater proportion than has been previously reported in some studies [11]. Likely candidates for other disease-associated polymorphisms in FMF are genes encoding proteins known to interact with pyrin or with a role in regulation of the IL-1β pathway [21]. Nevertheless, some studies have failed to identify an association between susceptibility to FMF and polymorphisms in genes involved in other autoinflammatory disorders [29] or suggested a modifying effect only on severity (e.g. NOD2/CARD15) [30]. These differences may relate to the specific allelic variants and ethnic populations studied.

Data from the Eurofever registry also point to an important role for environmental factors; for example, Eastern Mediterranean FMF patients have a milder disease phenotype once they migrate to Europe [31]. Limited data suggest that methylation status may alter *MEFV* expression [32], and recent pilot studies have identified several microRNAs expressed differentially in clinically quiescent FMF patients [33] and in patients with active disease [34] compared to healthy controls or healthy carriers [35]. Bidirectional cross-talk between gut microbiota and the systemic immune system may also affect expression of the disease [36]. More work is required to understand gene-environment interactions, the significance of epigenetic modifications, and their potential role as biomarkers.

In support of the hypothesis that modifying alleles have an additive or synergistic effect on the “total inflammatory burden” – as described in oligogenic autoinflammatory diseases – asymptomatic carriers of one FMF mutation may have biochemical evidence of subclinical inflammation [37]. Furthermore, patients who carry complex *MEFV* alleles appear to have more severe disease, suggesting an additive effect or cumulative burden of multiple mutations [38]. IL-1β production is also increased among carriers of high-penetrance *MEFV* mutations compared to healthy controls, with an intermediate phenotype in mononuclear cells from heterozygous patients [39], consistent with a decreased activation threshold for pyrin inflammasome activation [40]. A dose effect of *MEFV* mutations has also been suggested by studies on FMF animal models [8]. Moreover, the *MEFV* carrier state confers susceptibility to other inflammatory diseases including systemic onset JIA [41], adult-onset Still’s disease [42], sepsis [43], PFAPA [44, 45], Henoch-Schönlein purpura [46], polyarteritis nodosa [47], and Behçet disease [48]. Weak evidence that requires confirmation across multiple populations also suggests that *MEFV* mutations modify the course or severity of multiple sclerosis [49], Crohn’s disease [50, 51], and rheumatoid arthritis [52].

## An argument for biologically-informed treatment

Colchicine is the treatment of choice in FMF to reduce the severity and duration of symptoms as well as the risk of amyloidosis. However, 5–10% of patients show minimal response to colchicine and at least 30–40% continue to experience attacks [53]. Although generally safe and effective, colchicine’s narrow therapeutic window limits dose titration in patients with severe inflammatory phenotypes due to its toxicity. Furthermore, in complete responders, subclinical signs of inflammation such as elevated IL-18 levels may be present during afebrile periods [54]. Alternative treatment strategies include biologic agents targeting IL-1.

Biological therapies to treat FMF have been recently reviewed and include anakinra, an IL-1 receptor antagonist; rilonacept, a dimeric fusion protein that blocks the IL-1 receptor; and canakinumab, a human monoclonal anti-IL-1β antibody [55]. Recent studies in FMF patients with colchicine resistance have shown a significant reduction in attack frequency with anakinra or canakinumab [56, 57], with a complete response up to 16 weeks of ~ 70% for canakinumab [58]. Other agents that have been considered but with limited evidence to date include etanercept, a fusion protein targeting the tumour necrosis factor-α receptor; infliximab, a chimeric monoclonal antibody to TNF- α [59]; tocilizumab, a humanized monoclonal antibody targeting the IL-6 receptor [60]; and tofacitinib, a Janus kinase inhibitor [61]. Regardless of genotype, whether there is a dysregulated response to pro-inflammatory stimuli involving aberrant production of IL-1β or other cytokines can only be established by functional studies (e.g. analysis of enzyme activity, substrate binding/activation, subcellular localization, and cytokine release in response to inflammasome stimuli), which are currently not offered and may not be possible at all health care centres. In more difficult cases, ex vivo analysis of cytokine profiles may contribute not only to our understanding of disease pathogenesis, but also to identification of appropriate therapy. Ultimately, therapy for colchicine-resistant FMF will need to be tailored to individual patients based on our understanding of the pathways affected by their specific mutations.

In our experience, parents, government funding agencies, and insurers are often resistant to advocating for, and approving coverage for medications when a clinical diagnosis of FMF is not supported by a definitive pathogenic genotype. However, the risks of non-treatment in affected individuals may be significant; the most severe long-term complication of untreated severe FMF is systemic amyloidosis, typically affecting the kidneys and sometimes the adrenals, intestine, spleen, lung, heart, and thyroid [62]. Other sequelae can include recurrent pleural and pericardial effusions and infertility. Untreated patients may also develop splenomegaly, growth retardation, decreased bone density, and premature atherosclerosis [19]. Many patients at our centre who meet clinical diagnostic criteria for FMF do not meet criteria for starting colchicine based on recommendations for *MEFV* genotype analysis [13], either because of a single *MEFV* variant of undetermined significance or because of the presence of two such variants in *cis* (resulting in a complex allele). Our practical approach in treatment for these patients is to measure SAA levels between attacks, obtain urinalysis to monitor for the appearance of proteinuria, and recommend a therapeutic trial of colchicine for symptomatic patients, consistent with EULAR consensus recommendations for treatment as soon as a clinical diagnosis is made [63].

Most inclusion criteria for randomized controlled trials of biological therapies – including canakinumab, rilonacept, and anakinra – have required that patients be homozygous or heterozygous for pathogenic MEFV variants. This limits the available data for patients with unusual variants. Ultimately, clear guidelines will be required for access to drug coverage for patients with periodic fever syndromes – ideally incorporating the flexibility to tailor treatment to a patient’s unique biology. Coverage for biologic treatments for patients with periodic fever syndromes is limited across the country, and generally relies on having a specific identified genotype. Given our limited understanding of the genetic basis of these diseases, other objective measures based on cellular assays or inflammatory biomarkers, and including clinical features, may ultimately provide the best rationale for specific therapies.

## The importance of disease-focused databases

Research utilizing large numbers of clinical samples linked with relevant detailed clinical information is critical for targeting disease-specific biomarkers and validation of genetic risk factors of diseases, especially when studying gene variants with small effects. While initiatives began > 15 years ago in Europe to build the needed infrastructure for research in auto-inflammatory diseases [64], in Canada, our capacity to engage in research in this area is limited to a few centres that have the clinical expertise and resources for a local registry and/or biobank. It will be critical to develop population-based biobanks with a centralized longitudinal registry to support investigators and clinicians in their work. This is particularly important given our ethnically diverse population. Within 12 months of launch in 2017, 103 patients were enrolled in the BC Children’s Hospital Autoinflammation Disease registry, 65 of whom were diagnosed with a periodic fever syndrome (patients with chronic recurrent multifocal osteomyelitis excluded). This research registry is linked to a provincial Pediatric Autoinflammatory Diseases Clinic at the BC Children’s Hospital in Vancouver, BC where children and families can access diagnostic expertise and ongoing expert care. All patients diagnosed by one of the pediatric rheumatologists as having a PFS were eligible for entry into the registry. The most common diagnosis seen is “unclassified” PFS (*n* = 29) followed by PFAPA (*n* = 20) and FMF (*n* = 8). Genetic testing for at least one of the most common genes associated with monogenic disease has been offered to 56 patients with clinical suspicion for a given PFS. Results of genetic testing failed to classify 22 (39%) of these patients; 17 (30%) had no identified mutation in any of the known genes and 5 (9%) showed symptoms that were not consistent with the disease otherwise associated with that gene (Table 2). Our data is in line with results from the 2014 Canadian Periodic Fever Surveillance study in which the number of cases with unclassified PFS was reported to be as high as 40%, with 10% of these unclassified cases described as “FMF-like” based on clinical presentation [2]. These cases had clinical features of the specified PFS but either did not meet the full criteria or no confirmatory genetic testing was performed [2]. The heterogeneity of reported cases implies that genetic variations provide only a partial answer for disease etiology and highlights the importance of large cohort genotype/phenotype correlation studies. These above data also suggest that in multi-ethnic populations, making a clear diagnosis based only on gene variants is unsatisfying, making it difficult to offer appropriate treatment and prognostic information to families.

## Conclusions

A number of challenges exist in the diagnosis and management of FMF: (a) a single-gene recessive model of inheritance is inadequate for describing the spectrum of MEFV-associated phenotypes, particularly given the potential contribution of multiple genes in the complex pathways associated with cytokine synthesis; (b) interactions among multiple modifying alleles could give rise to a range of inflammatory phenotypes; (c) it is impossible to determine the significance of common variants in periodic fever genes in the absence of functional studies; and (d) access to biologic agents may be unjustly limited to patients with single allelic variants. More attention should be directed toward understanding the disease course and optimal therapy in patients heterozygous for *MEFV* mutations with variants of unknown significance. This will require ongoing international participation in collaborative registries such as the Eurofever Project and local efforts such as CAN-Fever and others, with collection of biological samples to allow investigation of potential susceptibility loci and modifier genes. To better understand these patients with inflammatory phenotype and unclear genotype results, we need to focus on functional biologic research to understand the significance of mutations with currently unknown function. Studies to validate data from molecular and clinical analyses will ultimately aid in tailoring therapy to patients with auto-inflammatory syndromes that lack a clear monogenic etiology, as well as discovery of new clinically relevant mutations.

## Abbreviations

FMF: Familial Mediterranean fever
IL-1: Interleukin-1
PFAPA: Periodic fever, aphthous stomatitis, pharyngitis and adenitis
PFS: Periodic fever syndrome
sJIA: Systemic-onset juvenile idiopathic arthritis
TRAPS: Tumour necrosis factor receptor-associated periodic syndrome

## References

[1] Manthiram K, Zhou Q, Aksentijevich I, Kastner DL (2017). The monogenic autoinflammatory diseases define new pathways in human innate immunity and inflammation. Nat Immunol.

[2] Dancey P, Besneler S, Gattorno M, Junker A, Laxer R, Miettunen P, et al. Surveillance of periodic fever syndromes in Canada [abstract]. Arthritis Rheumatol. 2015;67(S10).

[3] Ozen S (2018). Update on the epidemiology and disease outcome of familial Mediterranean fever. Best Pract Res Clin Rheumatol.

[4] Livneh A, Langevitz P, Zemer D, Zaks N, Kees S, Lidar T (1997). Criteria for the diagnosis of familial mediterranean fever. Arthritis Rheum.

[5] Yalcinkaya F, Ozen S, Ozcakar ZB, Aktay N, Cakar N, Duzova A (2009). A new set of criteria for the diagnosis of familial Mediterranean fever in childhood. Rheumatology (Oxford).

[6] Manukyan G, Aminov R (2016). Update on pyrin functions and mechanisms of familial Mediterranean fever. Front Microbiol.

[7] Sharma D, Malik A, Guy C, Vogel P, Kanneganti TD (2019). TNF/TNFR axis promotes pyrin inflammasome activation and distinctly modulates pyrin inflammasomopathy. J Clin Invest.

[8] Chae JJ, Cho YH, Lee GS, Cheng J, Liu PP, Feigenbaum L (2011). Gain-of-function pyrin mutations induce NLRP3 protein-independent interleukin-1beta activation and severe autoinflammation in mice. Immunity..

[9] Jamilloux Y, Lefeuvre L, Magnotti F, Martin A, Benezech S, Allatif O (2018). Familial Mediterranean fever mutations are hypermorphic mutations that specifically decrease the activation threshold of the pyrin inflammasome. Rheumatology (Oxford).

[10] Federici S, Sormani MP, Ozen S, Lachmann HJ, Amaryan G, Woo P (2015). Evidence-based provisional clinical classification criteria for autoinflammatory periodic fevers. Ann Rheum Dis.

[11] Ben-Chetrit E, Touitou I (2009). Familial mediterranean fever in the world. Arthritis Rheum.

[12] Gangemi S, Manti S, Procopio V, Casciaro M, Di Salvo E, Cutrupi M, et al. Lack of clear and univocal genotype-phenotype correlation in familial Mediterranean fever patients: a systematic review. Clin Genet. 2018. 10.1111/cge.13223 29393966

[13] Shinar Y, Obici L, Aksentijevich I, Bennetts B, Austrup F, Ceccherini I (2012). Guidelines for the genetic diagnosis of hereditary recurrent fevers. Ann Rheum Dis.

[14] Touitou I (2001). The spectrum of familial Mediterranean fever (FMF) mutations. Eur J Hum Genet.

[15] Ben-Chetrit E, Lerer I, Malamud E, Domingo C, Abeliovich D (2000). The E148Q mutation in the MEFV gene: is it a disease-causing mutation or a sequence variant?. Hum Mutat.

[16] Topaloglu R (2005). E148Q is a disease-causing MEFV mutation: a phenotypic evaluation in patients with familial Mediterranean fever. Ann Rheum Dis.

[17] Yasar Bilge NS, Sari I, Solmaz D, Senel S, Emmungil H, Kilic L (2018). Comparison of early versus late onset familial Mediterranean fever. Int J Rheum Dis.

[18] Cekin N, Akyurek ME, Pinarbasi E, Ozen F (2017). MEFV mutations and their relation to major clinical symptoms of familial Mediterranean fever. Gene..

[19] Ozen S, Bilginer Y (2013). A clinical guide to autoinflammatory diseases: familial Mediterranean fever and next-of-kin. Nat Rev Rheumatol.

[20] Toplak N, Frenkel J, Ozen S, Lachmann HJ, Woo P, Kone-Paut I (2012). An international registry on autoinflammatory diseases: the Eurofever experience. Ann Rheum Dis.

[21] Booty MG, Chae JJ, Masters SL, Remmers EF, Barham B, Le JM (2009). Familial mediterranean fever with a single MEFVmutation: where is the second hit?. Arthritis Rheum.

[22] Livneh A, Langevitz P, Zemer D, Zaks N, Kees S, Lidar T (1997). Criteria for the diagnosis of familial Mediterranean fever. Arthritis Rheum.

[23] Ben-Zvi I, Herskovizh C, Kukuy O, Kassel Y, Grossman C, Livneh A (2015). Familial Mediterranean fever without MEFV mutations: a case-control study. Orphanet J Rare Dis.

[24] Ben-Zvi I, Brandt B, Berkun Y, Lidar M, Livneh A (2012). The relative contribution of environmental and genetic factors to phenotypic variation in familial Mediterranean fever (FMF). Gene..

[25] Cazeneuve C, Ajrapetyan H, Papin S, Roudot-Thoraval F, Genevieve D, Mndjoyan E (2000). Identification of MEFV-independent modifying genetic factors for familial Mediterranean fever. Am J Hum Genet.

[26] Singh-Grewal D, Chaitow J, Aksentijevich I, Christodoulou J. Coexistent MEFV and CIAS1 mutations manifesting as familial Mediterranean fever plus deafness. ubcsummonserialssolutionscom. 10.1136/ard.2007.075655 PMC2111617 17934081

[27] Touitou I, Perez C, Dumont B, Federici L, Jorgensen C (2006). Refractory auto-inflammatory syndrome associated with digenic transmission of low-penetrance tumour necrosis factor receptor-associated periodic syndrome and cryopyrin-associated periodic syndrome mutations. Ann Rheum Dis.

[28] Ebadi N, Shakoori A, Razipour M, Salmaninejad A, Zarifian Yeganeh R, Mehrabi S (2017). The spectrum of familial Mediterranean fever gene (MEFV) mutations and genotypes in Iran, and report of a novel missense variant (R204H). Eur J Med Genet.

[29] Marek-Yagel D, Berkun Y, Padeh S, Lidar M, Shinar Y, Bar-Joseph I (2010). Role of the R92Q TNFRSF1A mutation in patients with familial Mediterranean fever. Arthritis Care Res (Hoboken).

[30] Berkun Y, Karban A, Padeh S, Pras E, Shinar Y, Lidar M (2012). NOD2/CARD15 gene mutations in patients with familial Mediterranean fever. Semin Arthritis Rheum.

[31] Ozen S, Demirkaya E, Amaryan G, Kone-Paut I, Polat A, Woo P (2014). Results from a multicentre international registry of familial Mediterranean fever: impact of environment on the expression of a monogenic disease in children. Ann Rheum Dis.

[32] Kirectepe AK, Kasapcopur O, Arisoy N, Celikyapi Erdem G, Hatemi G, Ozdogan H (2011). Analysis of MEFV exon methylation and expression patterns in familial Mediterranean fever. BMC Med Genet.

[33] Amarilyo G, Pillar N, Ben-Zvi I, Weissglas-Volkov D, Zalcman J, Harel L (2018). Analysis of microRNAs in familial Mediterranean fever. PLoS One.

[34] Hortu HO, Karaca E, Sozeri B, Gulez N, Makay B, Gunduz C, et al. Evaluation of the effects of miRNAs in familial Mediterranean fever. Clin Rheumatol. 2018. 10.1007/s10067-017-3914-0 29442258

[35] Akkaya-Ulum YZ, Balci-Peynircioglu B, Karadag O, Eroglu FK, Kalyoncu U, Kiraz S (2017). Alteration of the microRNA expression profile in familial Mediterranean fever patients. Clin Exp Rheumatol.

[36] Khachatryan ZA, Ktsoyan ZA, Manukyan GP, Kelly D, Ghazaryan KA, Aminov RI (2008). Predominant role of host genetics in controlling the composition of gut microbiota. PLoS One.

[37] Lachmann HJ (2006). Clinical and subclinical inflammation in patients with familial Mediterranean fever and in heterozygous carriers of MEFV mutations. Rheumatology..

[38] Gershoni-Baruch R, Brik R, Shinawi M, Livneh A (2002). The differential contribution of MEFV mutant alleles to the clinical profile of familial Mediterranean fever. Eur J Hum Genet.

[39] Omenetti A, Carta S, Delfino L, Martini A, Gattorno M, Rubartelli A (2014). Increased NLRP3-dependent interleukin 1beta secretion in patients with familial Mediterranean fever: correlation with MEFV genotype. Ann Rheum Dis.

[40] Jamilloux Y, Lefeuvre L, Magnotti F, Martin A, Benezech S, Allatif O, et al. Familial Mediterranean fever mutations are hypermorphic mutations that specifically decrease the activation threshold of the pyrin inflammasome. Rheumatology (Oxford). 2017. 10.1093/rheumatology/kex373 29040788

[41] Ayaz NA, Ozen S, Bilginer Y, Erguven M, Taskiran E, Yilmaz E (2009). MEFV mutations in systemic onset juvenile idiopathic arthritis. Rheumatology (Oxford).

[42] Sighart R, Rech J, Hueber A, Blank N, Lohr S, Reis A (2018). Evidence for genetic overlap between adult onset Still's disease and hereditary periodic fever syndromes. Rheumatol Int.

[43] Koc B, Oktenli C, Bulucu F, Karadurmus N, Sanisoglu SY, Gul D (2007). The rate of pyrin mutations in critically ill patients with systemic inflammatory response syndrome and sepsis: a pilot study. J Rheumatol.

[44] Di Gioia SA, Bedoni N, von Scheven-Gete A, Vanoni F, Superti-Furga A, Hofer M (2015). Analysis of the genetic basis of periodic fever with aphthous stomatitis, pharyngitis, and cervical adenitis (PFAPA) syndrome. Sci Rep.

[45] Berkun Y, Levy R, Hurwitz A, Meir-Harel M, Lidar M, Livneh A (2011). The familial Mediterranean fever gene as a modifier of periodic fever, aphthous stomatitis, pharyngitis, and adenopathy syndrome. Semin Arthritis Rheum.

[46] Altug U, Ensari C, Sayin DB, Ensari A (2013). MEFV gene mutations in Henoch-Schonlein purpura. Int J Rheum Dis.

[47] Yalcinkaya F, Ozcakar ZB, Kasapcopur O, Ozturk A, Akar N, Bakkaloglu A (2007). Prevalence of the MEFV gene mutations in childhood polyarteritis nodosa. J Pediatr.

[48] Wu Z, Zhang S, Li J, Chen S, Li P, Sun F (2015). Association between MEFV mutations M694V and M680I and Behcet's disease: a meta-analysis. PLoS One.

[49] Shinar Y, Livneh A, Villa Y, Pinhasov A, Zeitoun I, Kogan A (2003). Common mutations in the familial Mediterranean fever gene associate with rapid progression to disability in non-Ashkenazi Jewish multiple sclerosis patients. Genes Immun.

[50] Akyuz F, Besisik F, Ustek D, Ekmekci C, Uyar A, Pinarbasi B (2013). Association of the MEFV gene variations with inflammatory bowel disease in Turkey. J Clin Gastroenterol.

[51] Fidder H, Chowers Y, Ackerman Z, Pollak RD, Crusius JB, Livneh A (2005). The familial Mediterranean fever (MEVF) gene as a modifier of Crohn's disease. Am J Gastroenterol.

[52] Rabinovich E, Livneh A, Langevitz P, Brezniak N, Shinar E, Pras M (2005). Severe disease in patients with rheumatoid arthritis carrying a mutation in the Mediterranean fever gene. Ann Rheum Dis.

[53] Ozen S, Kone-Paut I, Gul A (2017). Colchicine resistance and intolerance in familial mediterranean fever: definition, causes, and alternative treatments. Semin Arthritis Rheum.

[54] Wada T, Toma T, Miyazawa H, Koizumi E, Shirahashi T, Matsuda Y, et al. Longitudinal analysis of serum interleukin-18 in patients with familial Mediterranean fever carrying MEFV mutations in exon 10. Cytokine. 2017. 10.1016/j.cyto.2017.10.007 29017770

[55] Ozen S, Demir S (2017). Monogenic periodic fever syndromes: treatment options for the pediatric patient. Paediatr Drugs.

[56] Akar S, Cetin P, Kalyoncu U, Karadag O, Sari I, Cinar M, et al. A Nationwide experience with the off-label use of Interleukin-1 targeting treatment in familial Mediterranean fever patients. Arthritis Care Res (Hoboken). 2017. 10.1002/acr.23446 28992387

[57] Ben-Zvi I, Kukuy O, Giat E, Pras E, Feld O, Kivity S (2017). Anakinra for colchicine-resistant familial Mediterranean fever: a randomized, double-blind, placebo-controlled trial. Arthritis Rheumatol.

[58] De Benedetti F, Gattorno M, Anton J, Ben-Chetrit E, Frenkel J, Hoffman HM (2018). Canakinumab for the treatment of autoinflammatory recurrent fever syndromes. N Engl J Med.

[59] Ozgocmen S, Akgul O (2011). Anti-TNF agents in familial Mediterranean fever: report of three cases and review of the literature. Mod Rheumatol.

[60] Ugurlu S, Hacioglu A, Adibnia Y, Hamuryudan V, Ozdogan H (2017). Tocilizumab in the treatment of twelve cases with aa amyloidosis secondary to familial mediterranean fever. Orphanet J Rare Dis..

[61] Gok K, Cengiz G, Erol K, Ozgocmen S (2017). Tofacitinib suppresses disease activity and febrile attacks in a patient with coexisting rheumatoid arthritis and familial Mediterranean fever. Acta Reumatol Port.

[62] Touitou I, Sarkisian T, Medlej-Hashim M, Tunca M, Livneh A, Cattan D (2007). Country as the primary risk factor for renal amyloidosis in familial Mediterranean fever. Arthritis Rheum.

[63] Ozen S, Demirkaya E, Erer B, Livneh A, Ben-Chetrit E, Giancane G (2016). EULAR recommendations for the management of familial Mediterranean fever. Ann Rheum Dis.

[64] Kuemmerle-Deschner JB, Hansmann S, Wulffraat NM, Vastert SJ, Hens K, Anton J (2018). Recommendations for collaborative paediatric research including biobanking in Europe: a single hub and access point for paediatric rheumatology in Europe (SHARE) initiative. Ann Rheum Dis.

